# Transformer-Based Topic Modeling: Characterizing Cannabis Product Adverse Experiences Self-Reported as Requiring Medical Attention on Reddit

**DOI:** 10.2196/82661

**Published:** 2026-02-04

**Authors:** Tim Ken Mackey, Matthew C Nali, Meng Zhen Larsen, Zhuoran Li, Cassandra L Taylor, Beverly Wolpert, Catharine Trice

**Affiliations:** 1 Global Health Program Department of Anthropology University of California, San Diego La Jolla, CA United States; 2 San Diego Supercomputer Center University of California, San Diego La Jolla, CA United States; 3 S-3 Research LLC San Diego, CA United States; 4 Global Health Policy and Data Institute San Diego, CA United States; 5 Center for Drug Evaluation and Research U.S. Food and Drug Administration Silver Spring, MD United States; 6 Office of Surveillance Strategy and Risk Prioritization Human Foods Program U.S. Food and Drug Administration College Park, MD United States; 7 Office of Women's Health U.S. Food and Drug Administration Silver Spring, MD United States

**Keywords:** cannabis, adverse events, reddit, machine learning, topic modeling

## Abstract

This study uses keyword filtering, a transformer-based algorithm, and inductive content coding to identify and characterize cannabis adverse experiences as discussed on the social media platform Reddit and reports a total of 1177 self-reported adverse experiences requiring medical attention.

## Introduction

Cannabis is one of the most frequently used intoxicating substances in the United States [1]. Changing policies and attitudes have increased cannabis use, yet safety profiles for different cannabis-derived products (CDPs) (eg, products containing tetrahydrocannabinols, cannabidiol, or cannabinoids derived from cannabis) are not well understood. Studies have examined internet and social media data for health concerns among cannabis users, including for adverse health outcomes [2-8]. However, no study has specifically characterized the types of adverse experiences requiring medical attention, as self-reported online. We aimed to use a transformer-based algorithm to identify and characterize adverse experiences self-reported as requiring medical attention (SRRMA) on Reddit.

## Methods

**Figure 1:**
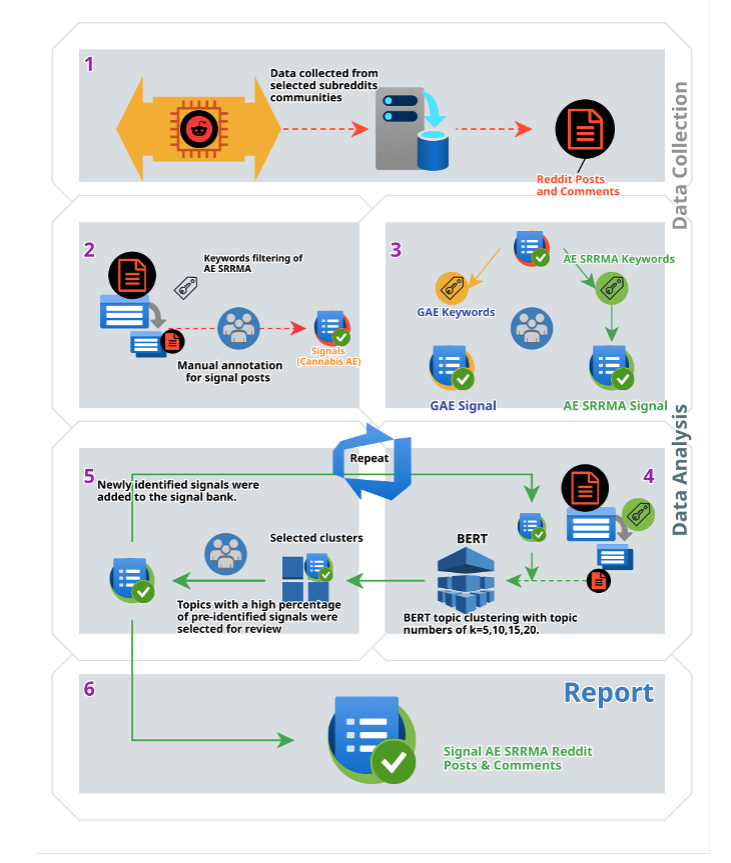
Summary of the study methodology, including data collection, data processing/filtering, BERT (bidirectional encoder representations from transformers) analysis, and results (signal data): (1) data collection for cannabis-related subreddits (both posts and comments); (2) keyword filtering for adverse experience self-reported as requiring medical attention (AE SRRMA)-related signals; (3) separation of sample of “signals” for general adverse experiences (GAE) self-reported as not requiring medical attention and AE SRRMA; (4) use of BERT topic model (k=5, 10, 15, 20) after data filtering with a sample of AE SRRMA keywords and labeled AE SRRMA data joined with the unlabeled dataset to analyze the full corpus of Reddit posts and comments; (5) selection of BERT topic clusters with the highest yield of prelabeled signals for AE SRRMA that were manually annotated to identify additional AE SRRMA signals (process repeated to achieve higher thematic saturation); newly identified signals were joined with the unlabeled dataset for the next round of BERT topic modeling; and (6) AE SRRMA signal identification from Reddit posts and comments summarized in this publication.

For this observational retrospective study, we collected user-generated posts and comments from 27 subreddits(/r/) related to cannabis use (Figure 1; Multimedia Appendix 1). A set of keywords for adverse experiences SRRMA was used for data mining and manual coding to generate a sample of “signal” Reddit data (ie, posts about cannabis product adverse experiences requiring medical attention: hospitalization, or visiting a health care professional, emergency room, or urgent care). This initial sample of SRMMA prelabeled “signal” was joined with data from the full Reddit data corpus.

Natural language processing was applied to cluster Reddit posts and comments for further manual annotation. We used the bidirectional encoder representations from transformers (BERT) topic model with the Python (version 3.7) package BERTopic (version 0.6.0), which is a pretrained, self-supervised transformer-based algorithm that embeds texts, extracts topics, and then clusters texts.

BERT was used to cluster groups of nonlabeled data with SRRMA prelabeled signal data into k topic clusters, where the value of k is determined according to the dataset size (5≤k≥20). In each generated cluster, we calculated the percentages of SRRMA prelabeled signal data in that topic cluster to estimate how well that cluster was related to our SRRMA prelabeled data (% of potential SRRMA prelabeled signal data in a cluster that could be appended to the SRRMA prelabeled dataset = amount of SRRMA prelabeled training signal data in a cluster/amount of total data in a cluster). The higher the percentage, the more likely the uncoded data were related to our SRRMA prelabeled topic. Cluster selection was determined based on the highest percentages. The new signals were then added to our coded signal pool for the next iteration of BERT modeling: we filtered the uncoded dataset with the keywords repeatedly, combined the filtered uncoded data with all prelabeled signal data, and then performed the next iteration of BERT to find the best clusters to manually annotate for identifying additional signals. Posts and comments from these clusters were extracted, and an inductive coding approach was used to label and characterize cannabis-related adverse experiences and adverse experiences that met the SRRMA criteria. Human ethics and consent to participate were not applicable, as all information was from the public domain and did not involve user interaction, and any identifiable information was aggregated and removed from the results.

## Results

We collected 1,795,478 Reddit posts/comments. After BERT and coding, 1177 posts/comments comprising 1542 user-generated mentions of SRRMA adverse experiences for cannabis products were detected between July 2017 and December 2022 (Table 1). Coders (TKM, MN, MZ) achieved a high intercoder reliability score (κ=0.90). From the 27 subreddits, the top 10 SRRMA adverse experiences were vomiting (284/1542, 18.42%), nausea (171/1542, 11.09%), panic attack (122/1542, 7.91%), abdominal/stomach pain (96/1542, 6.23%), concern over elevated heart rate (92/1542, 5.97%), anxiety (60/1542, 3.89%), chest pain (53/1542, 3.44%), general complaints of sickness (31/1542, 2.01%), symptoms attributed to Cannabinoid Hyperemesis Syndrome (30/1542, 1.95%), and paranoia (30/1542, 1.95%). Additionally, 108 (9.18%) SRRMAs mentioned a CDP but not a specific adverse experience. SRRMA adverse experiences included users self-reporting seeking medical attention with visits/admissions to hospitals (533/1177, 45.28%), emergency rooms (569/1177, 48.34%), health care professionals (70/1177, 5.95%), and urgent care (5/1177, 0.43%).

For cannabis product use characteristics with an SRRMA, most (988/1177, 83.94%) were inhalation (eg, inhaler, joint, vape) followed by ingestion (59/1177, 5.01%; eg, edibles) products. For users reporting intent of use (ie, post and comment), 94.65% (1114/1177) were adult use (ie, recreational), followed by 3.57% (42/1177) therapeutic (ie, for health benefit), 1.02% (12/1177) unknown, 0.42% (5/1177) medical (ie, from a medical dispensary), and 0.34% (4/1177) unintentional use. A subset of users reported co-use with other substances, specifically tobacco and nicotine products (n=7), prescription medications (eg, alprazolam, antidepressants, n=22), illicit drugs (eg, lysergic acid diethylamide, cocaine, n=5), and dual use with other cannabinoids (eg, general tetrahydrocannabinol use, cannabidiol, delta 8, n=12).

**Table 1:** Characteristics of adverse experiences self-reported as requiring medical attention, posted online on Reddit between July 2017 and December 2022.

Post and comment type/intent of use	Adverse Experiences Self-Reported as Requiring Medical Attention (AE SRRMA)^a^				
	Hospitalization (n=533), n	Health care professional visit (n=70), n	Emergency room visit (n=569), n	Urgent care (n=5), n	Proportion of platform AE SRRMA posts (N=1177), n (%)
Medical	1	0	4	0	5 (0.42)
Recreational	513	66	530	5	1114 (94.65)
Therapeutic	15	4	23	0	42 (3.57)
Unintended	0	0	4	0	4 (0.34)
Unknown	4	0	8	0	12 (1.02)

^a^AE SRRMA indicates that a cannabis user sought medical care because of a concern that their symptom or symptoms were serious or potentially life-threatening. Intent of use categories included medical (user mentions acquiring a product from a medical dispensary); recreational (user mentions using a cannabis product, price, or acquisition, in the context of a recreational or social event or circumstance); therapeutic (user mentions using a cannabis product for medical reasons); unknown (could not be determined based on data that was available); and unintended (user mentions using a cannabis-derived product accidentally or unintentionally).

## Discussion

We identified 1177 Reddit user-generated posts that described CDP SRMMA adverse experiences. Results are similar to those of a study analyzing 28,630 cannabis exposures/cases reported to state poison control centers from 2017 to 2019 that found CDP concentrates, vaporized liquids, and edibles documented with “moderate or greater” medical outcomes (ie, effects usually requiring a form of medical treatment) [9]. Studies using transformer-based and large language models also found similar reports of adverse experiences by Reddit users but only coded for generalized adverse health outcomes [7] or only reviewed a single subreddit (r/delta8) [8,9]. Our study builds on the methodology of these prior studies by using a transformer-based algorithm “seeded” with prelabeled signal data to find additional unlabeled data—an approach similar to a few-shot learning approach but without formal model training.

Study limitations include terms not inclusive of all cannabis safety-related experiences and lack of cross-validation of users’ self-reports (eg, lack of validation with clinical data). Further, social media data are not representative of all cannabis use behavior, and what cannabis products may have led to adverse experiences (eg, contaminated/adulterated products or products not from licensed dispensaries) were unclear (eg, we observed more Reddit discussions on adult-use cannabis [ie, recreational] than on medical cannabis use, which may limit generalizability). Though exploratory, study results may be used in conjunction with other cannabis-related adverse experience surveillance data (eg, National Poison Data System and FDA MedWatch [6]) and can provide important context to adverse experiences that result in medical attention for purposes of helping consumers and clinicians better understand potential cannabis risks.

## Appendix

Multimedia Appendix 1 Data collection, filtering, and content coding methodology.

## Abbreviations

BERT: bidirectional encoder representations from transformers
CDP: cannabis-derived product
SRRMA: self-reported as requiring medical attention
