# Supplementary material for: Transformer-Based Topic Modeling: Characterizing Cannabis Product Adverse Experiences Self-Reported as Requiring Medical Attention on Reddit
Source: J Med Internet Res. 2026 Feb 4;28:e82661. doi: 10.2196/82661 (PMC12917483; doi:10.2196/82661)
Supplement: Multimedia Appendix 1 [file jmir_v28i1e82661_app1.docx]

**Supplemental Online Content**

**eMethods.**

**eReference**

This supplemental material has been provided by the authors to give readers additional information about their work.

eMethods.

Data Collection

Data collection used a list subreddits associated with general cannabis discussions, use behaviors, and specific cannabis-derived products (CDPs) (see Appendix Item 1). We chose the social media platform Reddit based on its general popularity, accessibility of publicly available data at the time of the study, and different types of cannabis-related conversations. We used the Pushshift API and Python Reddit API wrapper package (PRAW Version 7.5.0) to collect Reddit data. Data collection commenced in February 2023 and completed in March 2023.

Data collected during the study time frame included retrospective data (posts, comments, reviews created by users prior to the data collection period that remained publicly available) and data created and collected during the data collection time frame that included a time period between January 2014 – December 2022. Study findings specific to Reddit posts and comments about adverse experiences (AEs) self-reported as requiring medical attention (SRRMA) occurred during the time frame between July 2017-December 2022. Underlying metadata (e.g., date and time at which the content was created, hyperlink to the content, title and text of the content, etc.) were also collected with all data exported to a password-protected database for manual content coding and further analysis. Data was de-identified to exclude specific usernames after collection and data cleaning.

Data Filtering

Keywords used for data filtering were chosen based on consideration of a list of medical and colloquial terms from the U.S. Food and Drug Administration’s MedDRA database, the published literature, and our own manual searches (see Appendix Items 2-3) with a focus on detecting user-generated content reporting firsthand (i.e., when a user reports directly experiencing an adverse experience) and secondhand experiences (i.e., when a user reports observations of other users or social contacts having an adverse experience) with cannabis products. Keywords were used independently to filter data to identify general adverse experiences and adverse experiences self-reported as requiring medical attention. Adverse experiences self-reported as not requiring medical attention were labeled as General Adverse Experience (GAE) and those self-reported as requiring medical attention were labeled Adverse Experience Self-Reported as Requiring Medical Attention (AE SRRMA). The study team chose the terminology (AEs) self-reported as requiring medical attention (SRRMA) as current Federal regulations defining “adverse events” and “serious outcomes” associated with adverse events generally apply to products meeting the definition of a drug or dietary supplement and most CDPs do not meet either definition.

Content Coding

The inductive content analysis approach included both a parent- and sub-classification coding schema that first identified if posts, comments, or reviews were relevant to the study aims specific to identification and characterization of adverse experiences self-reported as requiring medical attention (see Appendix Item 4 for coding schema). We excluded posts and reviews that did not appear to originate from actual users (e.g., organizational accounts, ads, bots, news agencies, etc.) or were not relevant to discussions about cannabis use and safety (e.g., cannabis product advertisements and promotional content, discussion about cannabis policy and legalization, etc.), which are hereinafter referred to as “noise.”

Parent classification of whether a post or review contained content related to a self-reported adverse experience used a binary coding classification of whether the content included a first-hand or second-hand user-generated report of an adverse experience associated with cannabis use, either in the course of use for medical purposes (e.g., medical marijuana use), for health benefit use (e.g., to purportedly treat a health condition), or adult-use (i.e., non-medical or recreational use).

In addition to the parent classification of whether the post or comment was adverse experience-related, content was also sub-coded for whether it constituted an AE SRRMA by assessing if one or more of the forms of medical attention identified [hospitalization, or healthcare professional, emergency room (ER), or urgent care visits] were reported. All content identified as an AE SRRMA was then coded verbatim for the specific adverse experience experienced (e.g., headache, respiratory distress, etc.) and the medical attention sought (e.g., death, hospitalization, disability, etc.).

Once a classification of whether a post or comment included a report of an AE SRRMA was made, content was further coded for other product and behavioral characteristics of interest: (a) cannabis product type associated with the adverse experience (e.g., edible, flower, vape device liquid, concentrate); (b) the route of administration of the product (i.e., the way in which a drug or substance is administered or taken into the body); (c) the intent of use of the product (e.g., what a user reports to be their reason to use cannabis, which can generally include medical use as accessed through a prescription or medical dispensary, for some claimed therapeutic use for health benefit, or adult-use); (d) the general or specific stated constituency of the cannabis product (e.g., product simply described as “marijuana”, products specifically mentioning cannabinoids, delta-8, or CBD; and (e) any mention of co-use with any other drug products or substances (including illicit substances).

Additional Results

Reddit Results: A total of 45 (3.82%) Reddit users also self-reported adverse experience SRRMAs and a specific health condition (e.g., celiac disease, high blood pressure, ulcerative colitis, etc.).

Other Adverse Experiences: This study focused on identification of adverse experiences self-reported as requiring medical attention; however, a high volume of posts and comments from Reddit included descriptions of various adverse experiences self-reported as not requiring medical attention.

eSupplement Appendix

Reddit: All available posts and comments were collected from selected subreddits

**Item 1. List of specific subreddits reviewed:**

“altcannabinoids”, “bostontrees”, “cannabiscultivation” “CHSexploration” CHSinfo”, “Dabs”, “delta8”, “delta8carts”, “Drugs”, “edibles”, “leaves”, “Marijuana”, “medicalcannabis”, “Michigents”, “MMJ”, “NewjerseyMarijuana”, “Petioles”, “QuittingWeed”, “Saplings”, “SousWeed”, “THCarts”, “thco”, “treedibles”, “trees”, “vaporents”, “weed”, “ZonaEnts”

**Item 2. List of Adverse Experience Keywords Used for Filtering Data:**

“Abuse”, “Addicted”, “Addiction”, “Aggravate”, “Agitation”, “Anorexia”, “Anxiety”, “Anxious”, “Arrhythmia”, “Blood”, “Blood Clot”, “Blood dycrasias”, “Blood pressure”, “Breath”, “Breathing”, “Bronchitis”, “Burn”, “Burning”, “Cancer”, “Cardiac”, “Chest pain”, “Chronic”, “Cognitive”, “Cold sweats”, “Coma”, “Complication”, “Condition”, “Consciousness”, “Constipation”, “Convulsion”, “Coronary, “Cough”, “Coughing”, “Damage”, “Depress”, “Depression”, “Diarrhea”, “Discomfort”, “Disease”, “Distress”, “Dizziness”, “Dizzy”, “Dyspnea”, “Elevated”, “Fainting”, “Gastrointestinal”, “Hallucinate”, “Hallucination”, “Headache”, “Heart”, “Heart Rhythm”, “Hurt”, “Hyper”, “Hypertension”, “Hyperthermia”, “Illness”, “Impaired”, “Infection”, “Inflammation”, “Inflamed”, “Injured”, “Injury”, “Intoxication”, “Irradic”, “Irritate”, “Irritated”, “Lungs”, “Lungs”, “Mediastinal”, “Mental health”, “Myocardial”, “Nervous system”, “Numb”, “Overdose”, “Pain”, “Palpitation”, “Panic”, “Panic Attack”, “Paranoia”, “Paranoid”, “Passed out”, “Passing out”, “Pneumonia”, “Poison”, “Poisoning”, “Psychological”, “Psychomotor”, “Pulmonary”, “Rash”, “Relax”, “Respiratory”, “Rhythmia”, “Sad”, “Schizophrenia”, “Scratch”, “Sedation”, “Seizure”, “Severity”, “Sick”, “Skin”, “Sleepy”, “Slowness”, “Slurred”, “Sore”, “Spasm”, “Stimulate”, “Sting”, “Stress”, “Stroke”, “Stung”, “Subcutaneous” ,”Syndrome”, “Thoracic”, “Threw up”, “Throat”, “Throw up”, “Thrush”, “Tingle”, “Tiredness”, “Tongue”, “Tremor”, “Ulcer”, “Uncoordinated”, “Vascular”, “Vomit”, “Vomiting”, “Weight loss”, “Wheeze”, “Wheezing”

**Item 3. List of Outcome Keywords Used for Filtering Data:**

“Allergic”, “Bedridden”, “Birth”, “Birth defect”, “Defect”, “CCU”, “Congenital”, “Congenital anomaly”, “CPR”, “Dead”, “Death”, “Dependent”, “Disability”, “Doctor”, “Drug Abuse”, “Drug Dependency”, “Emergency Room”, “Emergency-R”, “ER”, “E-Room”, “Home care”, “Hospital”, “Hospitalization”, “ICU”, Incapacity”, “Inpatient”, “Intensive”, “Intensive Care Unit”, “Life Support”, “Life-threatening”, “Persistent”, “Threatening”, “Withdrawal”, “Withdrawals”

Item 4: Coding Schema

| **Code Feature** | **Recorded Code** | **Code Description** |
| --- | --- | --- |
| Types of adverse experiences | Reported adverse experience | Type(s) of adverse experience user self-reported |
|  | N/a | No adverse experience mentioned |
| Adverse experience self-reported as requiring medical attention | Hospitalization | Caused user to be hospitalized |
|  | Emergency room | Caused user visit Emergency room |
|  | Healthcare Professional Visit | Caused user to visit a healthcare professional. |
|  | Urgent Care | Caused user to visit an urgent care facility |
| Health condition reported | Yes (Type of health condition) | Type(s) of health condition(s) user self-reported while using cannabis. |
|  | N/a | No mention of comorbidities self-reported by users of cannabis |
| Product type/Dosage form | Edible | Cannabis that is consumed orally |
|  | Drinkable | Cannabis beverages that are consumed orally |
|  | Capsule | Cannabis product made into capsules to be swallowed |
|  | Syringe | Cannabis product (liquid) delivered through a syringe application |
|  | Vape | Vapes that only use cannabis to inhale cannabis vapor |
|  | Joint | Weed hand-rolled to be used as combustible |
|  | Inhaler | Using inhaler device to deliver cannabinoids |
|  | Flower | Unprocessed form of cannabis (weed, pot) |
|  | Concentrate | Produced by distilling certain parts of the cannabis plant, can be consumed in different ways |
|  | N/a | No mention of product type being used |
| Strain type | Indica | User mentioned that the product contained Indica strain |
|  | Sativa | User mentioned that the product contained Sativa strain |
|  | Hybrid | Product or user mention hybrid strain was used |
|  | N/a | No mention of strain type |
| Co-use | Co-occurrence of Other Product Use with Cannabis | User self-reports co-using multiple products (tobacco, alcohol, etc.) |
|  | N/a | No self-reported co-use mentioned. |
| Routes of administration | Ingestion | Product consumed by eating/drinking. This can be in the form of edibles or oral means |
|  | Inhalation | Cannabis users mention they use a product by smoking, lighting up, or inhaling. |
|  | Multiple | User mentions that the product can be administered in multiple ways (e.g., inhalation, ingestion, etc.). |
|  | Unknown | Cannabis user does not mention how they are using a product |
| Intent of use | Medical-use | User mentions acquiring a product from a medical dispensary or possession of a medical marijuana card |
|  | Therapeutic-use | User mentions using a product for health benefit reasons but does not mention acquiring the product from a medical dispensary |
|  | Adult-use (recreational) | User mentions terms related to recreational or casual use and does not mention use in the context of medical or health benefit purposes |
|  | Unintended | User mentions using a CDP accidentally or unintentionally |
|  | Unknown | User does not mention why they are using a product and/or unable to make a connection that a CDP is being used for adult-use purpose |

Item 5: Breakdown by Post and Comments for Characteristics of Adverse Experiences Self-Reported as Requiring Medical Attention, Posted Online on Reddit Between July 2017-December 2022

| **Item 5: Characteristics of Adverse Experiences Self-Reported as Requiring Medical Attention, Posted Online on Reddit Between July 2017-December 2022** | | | | | | | | | |
| --- | --- | --- | --- | --- | --- | --- | --- | --- | --- |
| **Type**  **(n, % of Total)** | | **Intent of Use** | **Adverse Experiences Self-Reported as Requiring Medical Attention (AE SRRMA)** | | | | | | |
|  |  |  | **Hospitalization** | **Healthcare professional visit** | **ER visit** | **Urgent Care** | **Total** | **% of platform AE SRRMA posts** |  |
| Reddit | Post  (n=1,115, 94.73%) | Medical | 1 | 0 | 4 | 0 | **5** | **0.45%** |  |
|  |  | Recreational | 496 | 57 | 497 | 5 | **1,055** | **94.62%** |  |
|  |  | Therapeutic | 15 | 4 | 23 | 0 | **42** | **3.76%** |  |
|  |  | Unintended | 0 | 0 | 4 | 0 | **4** | **0.36%** |  |
|  |  | Unknown | 2 | 0 | 7 | 0 | **9** | **0.81%** |  |
|  | **Total** |  | **514** | **61** | **535** | **5** | **1,115** | **100%** |  |
|  | Comment  (n=62, 5.27%) | Medical | 0 | 0 | 0 | 0 | **0** | **0.00%** |  |
|  |  | Recreational | 17 | 9 | 33 | 0 | **59** | **95.16%** |  |
|  |  | Therapeutic | 0 | 0 | 0 | 0 | **0** | **0.00%** |  |
|  |  | Unintended | 0 | 0 | 0 | 0 | **0** | **0.00%** |  |
|  |  | Unknown | 2 | 0 | 1 | 0 | **3** | **4.84%** |  |
|  | **Total** |  | **19** | **9** | **34** | **0** | **62** | **100%** |  |
| **Total for all Post/Reviews** | |  | **533** | **70** | **569** | **5** | **1,177** |  |  |
| **Definitions:** *Adverse experiences self-reported as requiring medical attention (SRRMA) indicate that a cannabis user sought medical care because of a concern that their symptom or symptoms were serious or potentially life-threatening. Intent of use categories included medical (user mentions acquiring a product from a medical dispensary); recreational (user mentions using a cannabis product, price, or acquisition, in the context of a recreational or social event or circumstance); therapeutic (user mentions using a cannabis product for medical reasons); unknown (could not be determined based on data that was available); and unintended (user mentions using a CDP accidentally or unintentionally).* | | | | | | | | |  |
